# HIV, Stigma, and Rates of Infection: But Is AIDS-Related Stigma Understood?

**DOI:** 10.1371/journal.pmed.0040053

**Published:** 2007-01-30

**Authors:** Arachu Castro

Although its title attracted me to read this paper [1], I was quickly disappointed. First, it advances a Malthusian approach that applauds the “potential benefits” of AIDS-related stigma. Second, it reflects a poor understanding of some of the existing literature on AIDS-related stigma cited in the article. The authors claim that the idea that stigma and discrimination thwart efforts to control the epidemic and constitute barriers for prevention and treatment is said over and over, with no evidence, “like a shibboleth,” and cite eleven references that are meant as examples of this uncritical repetition. Surprisingly though, at least one of the articles cited already argues that “confusion surrounds debate over stigma as a barrier to introducing antiretrovirals to poor countries or to making voluntary HIV tests accessible” and that “to assess AIDS-related stigma and declare it a cause rather than both cause and consequence of inequality will probably weaken efforts to address AIDS among those with heightened risk of HIV because of poverty, racism, and gender inequality” [2]. Finally, the authors show limited understanding of social and sexual dynamics. Their distinction between “subpopulations” and “the general population” seems to imply that the former, such as the subpopulation of sex workers, lives in isolation of the latter and that it takes a certain amount of time for HIV to go from one to the other; it implies, for example, that sex workers first transmit HIV amongst themselves and then at some later point they transmit it to “the general population.” I am afraid that the authors are actually conceptualizing HIV transmission within the similar linear frameworks that they are trying, unsuccessfully, to criticize.
